# Visual Attention during Spatial Language Comprehension

**DOI:** 10.1371/journal.pone.0115758

**Published:** 2015-01-21

**Authors:** Michele Burigo, Pia Knoeferle

**Affiliations:** Department of Linguistics, University of Bielefeld, Bielefeld, Germany and Language & Cognition Research Group, Cognitive Interaction Technology—Center of Excellence (CITEC), University of Bielefeld, Bielefeld, Germany; University of Verona, ITALY

## Abstract

Spatial terms such as “above”, “in front of”, and “on the left of” are all essential for describing the location of one object relative to another object in everyday communication. Apprehending such spatial relations involves relating linguistic to object representations by means of attention. This requires at least one attentional shift, and models such as the Attentional Vector Sum (*AVS*) predict the direction of that attention shift, from the sausage to the box for spatial utterances such as “The box is above the sausage”. To the extent that this prediction generalizes to *overt* gaze shifts, a listener’s visual attention should shift from the sausage to the box. However, listeners tend to rapidly look at referents in their order of mention and even anticipate them based on linguistic cues, a behavior that predicts a converse attentional shift from the box to the sausage. Four eye-tracking experiments assessed the role of overt attention in spatial language comprehension by examining to which extent visual attention is guided by words in the utterance and to which extent it also shifts “against the grain” of the unfolding sentence. The outcome suggests that comprehenders’ visual attention is predominantly guided by their interpretation of the spatial description. Visual shifts against the grain occurred only when comprehenders had some extra time, and their absence did not affect comprehension accuracy. However, the timing of this reverse gaze shift on a trial correlated with that trial’s verification time. Thus, while the *timing* of these gaze shifts is subtly related to the verification time, their *presence* is not necessary for successful verification of spatial relations.

## Introduction

In everyday communication we are exchanging spatial information about our environment via what has been dubbed *spatial language*. For instance, in the spatial description “The box is above the sausage”, the location of the *sausage* (the reference object) [1], [2] narrows the domain in which to search for the *box* (the located object) [3], [4]. The spatial preposition “above” defines the relation between the sausage and the box. While we know that its interpretation engages attentional resources [5], [6], we do not know how precisely visual attention is deployed during the online comprehension of sentences about spatial relations. This is because most studies on spatial language processing have focused on *covert* attention shifts in response to spatial prepositions (e.g., [7], [8], [9]). Other studies have examined *overt* visual attention shifts but only during scene inspection after sentence comprehension [10]. And in the few eye-tracking studies in which participants inspected a scene as they listened to spatial descriptions, their visual attention was not analyzed in great detail (of which more below, [11]). The present paper, by contrast, uses eye tracking to assess how precisely *overt* visual attention is deployed during spatial language comprehension. The interest towards overt attention is motivated mainly by the observation that language comprehension is an incremental process and eye movements provide a measure of how this process unfolds [12], [13]. Overall, eye movements have a central role in language processing research [14] and our goal is to better understand their role in the comprehension of spatial descriptions.

## Covert Attention and the Processing of Spatial Relations

A range of findings in the literature supports the view that covert attention is involved in the comprehension of spatial relationships [6], [9], [15], [16]. In a visual search task, participants detected a target object pair (e.g., a plus above a dash) faster when it differed from a distractor pair in its elements (e.g., a plus above a plus) than in its spatial relation (e.g., below vs. above a dash); moreover, when the difference concerned the spatial relation, target search time increased proportional to the number of distractors [6]. This response-time increase suggests that discriminating between object pairs based on their contrastive spatial relations requires attentional resources. In a related experiment, participants reported whether one circle was spinning inside or outside another (concentric) circle. When the two circles were spinning slowly, judging their relative spatial position was possible but as their speed increased, the participants made an increasing number of errors. This decline in performance was taken as evidence that processing spatial relationships requires selective attention to objects [8].

In another study, participants judged the spatial relation of differently colored geometric shapes (e.g., whether a magenta diamond was to the left of a green diamond). To detect shifts in spatial attention, Franconeri and colleagues recorded event-related brain potentials (ERPs) and examined a posterior negativity (N2Pc), contra-lateral to the attended visual field that had previously been associated with shifts in attention (e.g., see [17], [18]). Negative polarity indicated a shift of attention from the central fixation point towards (an object) on one side of the screen, and positive polarity indicated a shift to the other side of the screen. Their analysis showed that the ERP signal went from negative polarity shortly after stimulus presentation (200–300 ms) to positive polarity (peak at approximately 400 ms). This polarity change was interpreted as evidence for the view that participants systematically shifted covert attention across the to-be-judged geometric shapes while maintaining their gaze steady on a fixation mark [7].

The implication of attention in spatial-relation processing has received further support from a change detection task in which the change concerned either the spatial relation (“A above B” became “A on left of B”) or another aspect of the visual context (e.g. object distance, presence/absence). Participants detected the change faster when it concerned spatial relations, and faster when the target location was visually cued (vs. not cued). The difference in response times between the spatial-relation and the distance condition, however, did not vary as a function of whether attention was (vs. wasn’t) cued to the target location [9]. These results were taken to mean that the spatial relations of objects are not encoded pre-attentively but rather once spatial attention has been allocated to the target location.

The observed data pattern [7], [8] reflects *covert* attention shifts: In the studies by Franconeri et al., trials with overt eye movements were eliminated; Holcombe et al. did not monitor eye gaze but argued that their participants had extensive experience in maintaining fixation and could be trusted to keep their eyes on the fixation point. In Rosielle et al., participants were allowed to move their eyes, and they were faster to detect changes in the spatial relation than in the distance of objects both when their visual attention was and when it wasn’t guided to the target. This suggests that guiding overt visual attention to the target was not essential in the response time advantage. However, Rosielle et al. did not monitor the deployment of visual attention and their task did not involve language. In sum, these results [7], [8], [9], cannot provide insights into how *overt* visual attention is deployed during the comprehension of sentences about spatial relations.

## Overt Attention and the Processing of Spatial Relations

Some initial evidence on the role of overt attention in spatial language comprehension comes from visual-world studies. In these studies, participants’ eye movements are monitored as they listen to spoken utterances and inspect a related real or semi-real visual context. Participants tend to more often inspect mentioned than unmentioned objects (e.g., [19], [12]) and they anticipate potential linguistic reference to objects (e.g., [20], [21]) even when speech is rapid and scenes are cluttered [22]. With regard to spatial language, prepositions such as “into” in “Put the whistle into…” rapidly guide listeners’ visual attention to objects that could serve as a container for the whistle (e.g., a can large enough to contain the whistle, [11]). In a similar study participants were verbally instructed to move playing cards above or below other objects (e.g. “Put the five of heart that is above the ten of clubs, below the King of spades”, [23]). When “above” disambiguated reference to the five of heart (only one card was above the ten of clubs), listeners inspected that card shortly after disambiguation through “above” and earlier than when disambiguation occurred at “ten”, or at the last word (“spades”). In summary, spatial terms are interpreted incrementally and constrain visual attention to relevant referents during spoken language comprehension.

**Figure 1:**
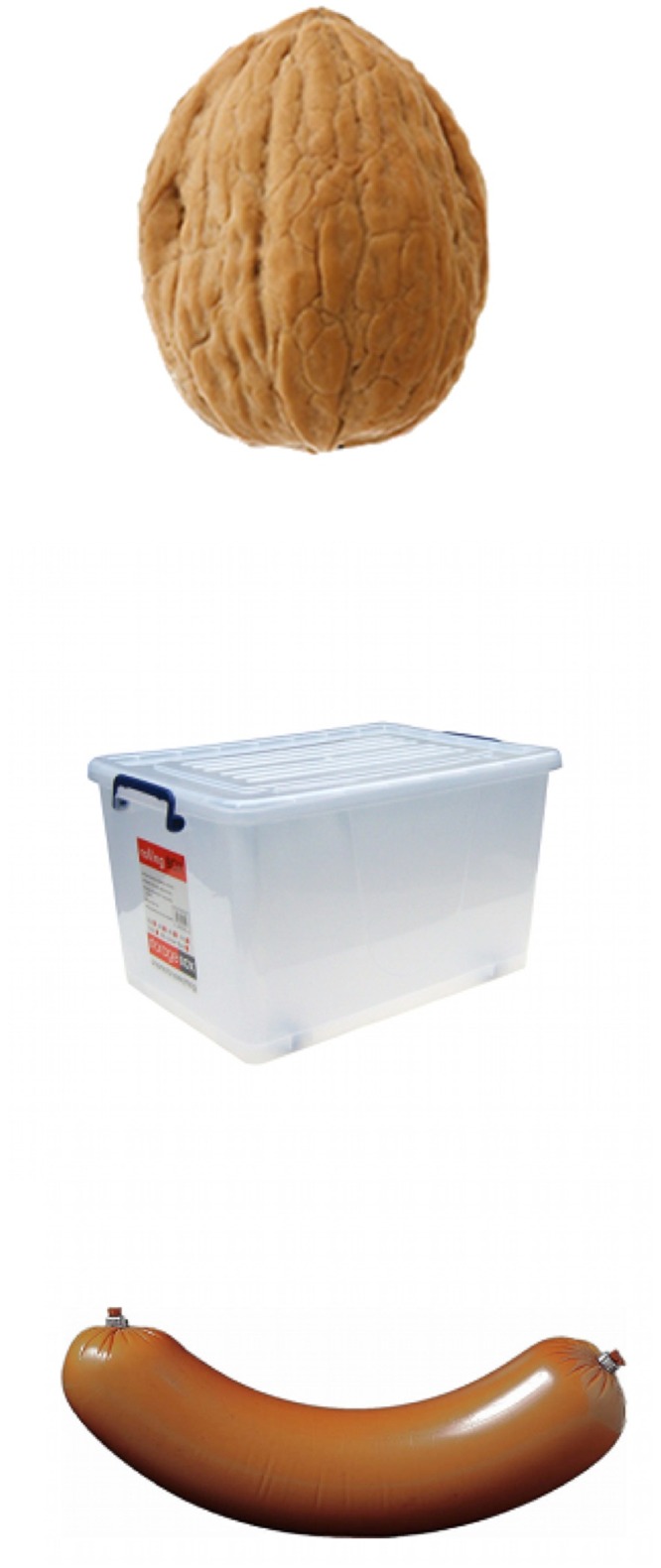
Example scene for the sentence “Die Box ist über der Wurst”, ‘The box is above the sausage’. The box is the object whose location has been described (the so called “located object”) and the sausage is the object acting as a reference point (“the reference object”). The walnut is a distractor; it is not mentioned in the sentence but may capture some attention before disambiguation through the spatial preposition (“the competitor object”).

One model that can further inform us about attention in response to spatial prepositions is the Attentional Vector Sum model (AVS, [24]). The model simulates the attentional processes implicated in understanding the kinds of spatial relations that have been examined in visual-world studies (e.g., [11]). The direction of attention in the model is simulated by the activity of a population of neurons, and summarized by a representative vector. According to the model, the averaged direction of this vector must go from the reference object (the sausage) to the located object (the box, in “The box is above the sausage”, see also Fig. 1). There is reason to believe that an orientation-tuned population of neurons (such as the ones simulated in the model) predicts the direction of a *motor action* [25], including eye movements. To the extent that this prediction extends to overt eye movements, we should see an overt gaze shift from the sausage to the box. Similar assumptions have been made elsewhere, viz. that an attentional shift from the reference to the located object is critical for understanding a spatial relation [26] and that this attention shift can be reflected in overt gaze shifts ([10], p. 203).

The direction of the attention shift predicted by the AVS model goes against the grain of incremental comprehension (from the second- to the first-mentioned object). Comparing the AVS-motivated predictions with the pattern predicted by the visual-world results (the converse, viz. anticipating the sausage after hearing “The box is above…”) has—to the best of our knowledge—not yet been done and is not straightforward. This is because the AVS model makes no predictions about the time course of spatial language comprehension. What it does predict, however, is that we can only understand the spatial term “above” by shifting attention from the sausage to the box. If this is true, then it seems reasonable to assume that this shift should occur shortly after comprehenders heard “above”. The visual-world studies, by contrast, make a clear time-course prediction whereby comprehenders should anticipate the sausage shortly after hearing “above” in “The box is above….”.

In this paper, we present the results from four eye-tracking studies which examined visual attention during sentence comprehension immediately after the spatial preposition (“Die Box ist über…”, ‘The box is above…’). Our goal was to assess, for the first time, the predictions motivated by the AVS model for overt gaze shifts during spatial language processing. To further gain insight into the functional significance of any shifts from the reference to the located object, we varied participants’ task and the presence of the mentioned objects across the four studies (of which more below). In summary, we observed the shift predicted by the AVS model in overt eye movements, but that shift was infrequent, suggesting it is not mandatory for accurate verification of spatial descriptions. On average, the gaze pattern matched the anticipation of upcoming referent locations (see [11]). The attentional shifts back to the located object (the box) following “above” were further modulated by the task and by object presence, and the timing of such a shift on a trial correlated with the timing of that trial’s verification response.

## Experiment 1

### Method

#### Participants

Thirty-two students (average age = 23, range = 19–33) from the University of Bielefeld received five euro each for taking part in this study. All participants had normal or corrected-to-normal vision, were native speakers of German, and monolingual before age 6.

#### Ethics statement

The experimental procedure was approved by the Bielefeld University ethics committee (Nr. 2014–060). Participants read an Information sheet containing general information about the experiment and data treatment. They were then asked to sign an Informed Consent form. At the end of the experiment, participants were debriefed and had the possibility to ask questions about the experiment.

#### Materials and design

The experiment included 32 critical trials and 60 fillers (92 trials). Each scene included three objects: the located object (e.g., the box in Fig. 1), the reference object (e.g., the sausage) and a competitor object (e.g., the walnut). The located object and the reference object were mentioned in the sentence. We included the third, unmentioned competitor object to prevent participants from guessing the reference object before the onset of the spatial term.

**Figure 2:**
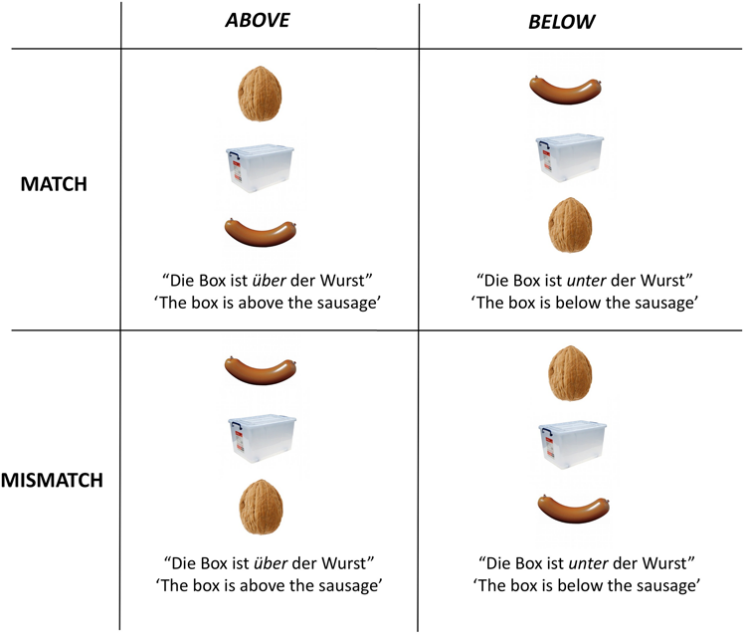
Summary of the experimental design. The manipulated factors were: 2 sentence values (match vs. mismatch) x 2 spatial prepositions (“über” (‘over’) vs. “unter” (‘below’)). Mismatch trials were included to give participants a clear task and will not be examined for the eye-movement analyses; but they will be examined in the response time analysis. Trials with different spatial prepositions were included for counterbalancing reasons and we collapsed across preposition in the analyses.

The design included 2 factors with 2 levels each: spatial preposition (“über”, ‘above’ vs. “unter”, ‘below’) and sentence value (match condition: objects displayed according to the spatial description vs. mismatch condition: objects not displayed according to the spatial description, see Fig. 2). Mismatch trials were included in order to have a balanced design and we only included them in the analysis of verification time to see if we could replicate the match/mismatch effect observed in response times [27], [28]. In the eye-movement analyses, we included only matching trials.

**Figure 3:**
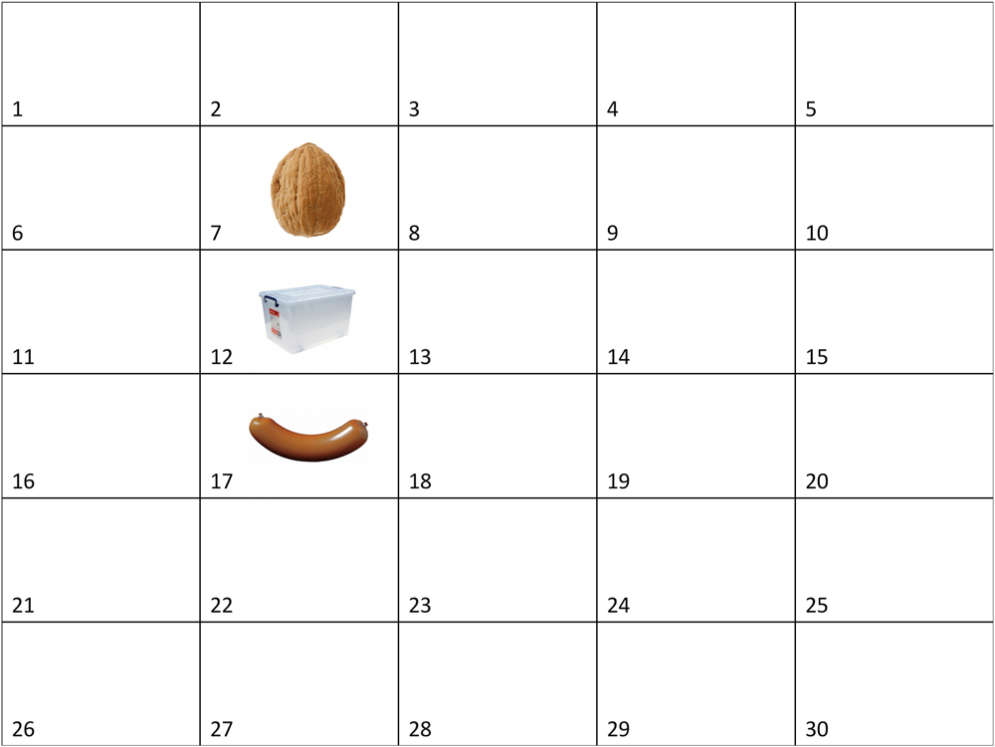
Example scene. This figure illustrates the 5x6 grid used in all the experiments to allocate object position. The grid and its numbers were invisible to participants.

The three objects of an item were always shown vertically aligned. Object locations were based on a 5 x 6 virtual grid (numbered from 1 to 30 starting from the top left square; see Fig. 3). Because the edges of the monitor were close to the trackable range of the eye tracker (SR Research Ltd., 2010), objects were never shown in the top- and bottom-most rows (squares 1 to 5 & 26 to 30, Fig. 1) in order to keep tracking accuracy high. The resulting four sets of cells in which the objects appeared were: set 1 = cells 7–12–17, set 2 = cells 9–14–19, set 3 = cells 12–17–22 and set 4 = cells 14–19–24 (Fig. 3 illustrates set 1). Spoken sentences presented with the scenes were in German and had the following format: [located object] [verb] [spatial preposition] [reference object], whereby the spatial prepositions could be “über” (‘above’) or “unter” (‘under’) (see Section E in S1 File for the full list of sentences).

Given that functional relations between the objects can influence the way people process spatial descriptions [29], we presented a total of 350 object pairs to participants (N = 17) and asked them to rate the probability that the two objects in each pair interact with each other (1 = low probability to 7 = very high probability). A violin and a violin bow, for instance, would often interact, and receive a high rating (e.g., 6 or 7). In contrast, a violin and a window typically do not interact and would receive a low rating (e.g., 1 or 2). Pairs of objects with a rating above 3 (N = 24) were excluded from the experiment. The object names in all of the pairs were controlled for the number of syllables, article gender, and frequency (all *t*s < 0.12). From the remaining items, we created 32 triplets based on low average functionality ratings for pairs (e.g., a window and a violin; a window and a flower were used to create a triplet with a window, a violin and a flower). The pictures were resized in a 300 x 300 pixel format on a white background.

For sixteen filler trials the sentence referred to a reference object that was not in the picture (e.g., “Die Puppe ist über dem Lenkrad”, ‘The doll is above the steering wheel’ with a figure showing a harp, a doll, and a funnel; 1/5 of all trials). This discourages people from deciding about the correct placement of the located object based on its absolute location (or in reference to the computer monitor) resulting in anticipated answers and no fixations to the reference object [30], [31]. The other filler sentences (44 trials) included different types of sentence structures and other spatial prepositions such as “zwischen” (‘between’), “nahe bei” (‘near’), “um herum” (‘around’). Item-condition combinations were assigned to the experimental lists following a Latin square design. Each participant saw only one version of an item, and the same number of trials for each condition.

#### Procedure

An EyeLink 1000 (SR Research Ltd., Ontario, Canada) with a desktop mount monitored participants’ eye movements at a frequency of 1000 Hz. Eye gaze and reaction times were recorded using the stimulus presentation software SR Research Experiment Builder (version 1.10.165). Participants were seated at approximately 85 cm from the screen (22-inch color monitor; 1680x1050 pixel resolution) with their chin on a chin-rest. Before the experiment, participants read the instructions and nine practice trials familiarized them with the procedure. Participants were instructed to try to understand the sentences and to attentively inspect the image, and to respond per button press as quickly and accurately as possible whether the sentence matched (vs. didn’t match) the picture (even before the end of the sentence). The experimenter performed a calibration of the eye tracker at the start of the experiment, and after half of the 92 trials. On each trial, participants fixated a circle in the middle of the screen, permitting the eye tracker to perform a drift correction if necessary. Then another fixation point appeared centrally for 1500 ms, after which the picture and the sentence were presented. Given the illusionary delay people experience at scene onset [32], we included a 750 ms preview. An ISI of 2500 ms ended the trial. Calibration took approximately 5 minutes, and the experiment lasted around 30 minutes.

### Analyses

#### Response times

For the analysis of response times we modeled the RT distribution with a Linear Mixed Effects Regression (LMER) including items and participants as random factors [33]. We used R (R development core team [34]), and the *lme4* package [35], [36], which provided a reliable algorithms for parameter estimation. Following Barr and colleagues [37], we first computed the most complex converging model, and then performed a stepwise reduction based on model comparison in order to capture the “maximal random effect structure justified by the data” [38]. We compared the most complex model with a model which excludes the factor with the lowest variance (i.e., we are removing elements that contribute little towards explaining the variance of the entire model). This procedure continued until the removal of a factor led to a significant decrease in model fit (log-likelihood ratio) or until the model contained only main effects (see Table A in S1 File) for a summary of all models used). The resulting model is the “final” model for which we report *t-*values and SE for all fixed effects and interactions, if present.

Only trials with a correct response were included, and predictors were centered in order to prevent statistical inferential errors [39]. The effect of extreme values was minimized through log transformation and through the use of random effect terms in the LMER (these adjust the average latency by means of small changes to the intercept, see [33]). In the RT analysis we included match and mismatch trials because we were interested in replicating the advantage for matching responses observed in previous studies [27], [28].

#### Eye movements

Fixations that started before a critical time window and that ended within it, as well as fixations < 80 ms were removed prior to further analysis. For the eye-movement analysis we collapsed across spatial prepositions (“über”, ‘above’ vs. “unter”, ‘below’)—first because this factor is irrelevant in contrasting the AVS-based with the visual-world-based expectations and second because it is known that both prepositions guide attention [5] and only differ in the direction of the attentional shift. We also excluded data from mismatching trials (included to see if we replicate the established congruence effect in the RTs), since the AVS account makes no clear prediction about incremental mismatch processing.

The visual scene was divided into 4 Areas of Interest (AoIs): one for each of the three objects and the background. Regions for the object had the same size as the original picture, 300 x 300 pixels, and were coded as located object, reference object (the two “targets”), and competitor respectively. We were interested in examining visual attention immediately after the spatial preposition and after at least one inspection had been directed towards the reference object. This “condition” was necessary to ensure the eye-movement patterns have a shared starting point that defines when the visual-world results and the AVS account suggest diverging shifts in visual attention. Thus, we focused on a time window from the onset of the spatial preposition (+200 ms) to the response and examined the first four inspections to the AoIs after one reference object inspection. For more complete analyses of the eye-movement pattern across the sentence, see Section B in S1 File. Inspections by definition capture a shift of gaze to another region and hence the first inspection after leaving the reference object must be directed towards either the located object or the competitor object. Accordingly, this analysis will include only trials on which this gaze pattern manifests itself.

Recall that people tend to anticipate the post-preposition object shortly after a spatial preposition (e.g., the sausage in Fig. 1). We were interested in what happened next: According to the findings from visual-world studies participants should continue to fixate the reference object as it is mentioned. By contrast, the AVS model predicts that attention shifts from the reference object (the sausage) to the located object (the box) and people should be more likely to inspect the located object than the reference object after the spatial preposition. This shift in attention is not predicted by the visual-world results.

We divided the probability of inspecting the located object (LO) by the probability of looks to the reference object (RO): *ln*(P(LO)/P(RO)). We also computed the log gaze probability including the looks directed toward the competitor and to the background. Since these looks did not affect the log gaze probability pattern they were not analyzed further. The resulting log-gaze probability ratio is suitable for examining inspection preferences (see [40], [41], [42]). A zero value in the log-gaze probability ratio indicates participants are equally likely to inspect the located and the reference object; a positive score indicates that they are more likely to inspect the located object than the reference object; and a negative value that they are more likely to inspect the reference object than the located object. The absolute value of the log-ratio reflects the magnitude of the effect (the intercept of the linear model). A significant intercept in the linear model indicates that the intercept differs from zero, revealing a preference for inspecting one of the two objects [42]. When the devisor in the log-ratio was 0 (no inspection to the reference object), we added a constant to both the nominator and denominator.

For each of the four inspections following the onset of the spatial preposition (+200 ms), and after one inspection had gone to the reference object, we calculated the log gaze probability ratio per subject; these log-gaze probabilities constituted the predictor in a linear model with four levels (inspections 1–4; all models are reported in Table A in S1 File). The predictor was centered (M = 0, SD = 1), and the intercept represents the grand mean of the log gaze probability coefficients. To also gain insight into how often in the course of the experiment people shifted from the reference to the located object we computed the number or trials on which this behavior occurred between spatial preposition onset (+200) and the response.

### Results

Trials with incorrect responses (3.1% of the data) were removed from the analysis.

#### Response times

The LMER model revealed a significant main effect of sentence value (t = 7.8, SE = 16.3) and of spatial preposition (t = 2, SE = 8.33). Responses were faster for matching (M = 5013; SD = 222) than mismatching trials (M = 5258; SD = 244), and faster for spatial descriptions with “über”, ‘above’ (M = 5120 ms; SD = 223 ms) than “unter”, ‘below’ (M = 5152 ms; SD = 236 ms). The interaction was not significant (t = 1.4, SE = 8.26).

#### Eye movements

**Figure 4:**
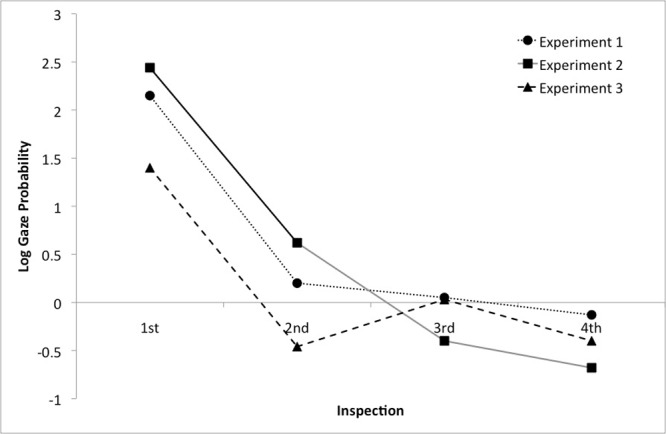
Log gaze probability of inspections. This graph illustrates the probability of the first four inspections towards the located object (positive values) and the reference object (negative values) for Experiments 1, 2 and 3. These were calculated for the interval between *Preposition onset* (+200 ms) and *Response* (only by-subjects data are displayed) and only after one inspection to the reference object occurred.

The analysis revealed a significant intercept (Estimated coefficient_ss_ = 0.76, t = 2.73, p < .01; Estimated coefficient_item_ = 0.87, t = 1.93, p = .055) with a positive value, suggesting that the inspections after once looking at the reference object were mostly directed towards the located object (see Fig. 4). However, the analysis on the number of trials revealed that this behavior was rare (N = 110, 21.8% of the 505 correctly answered experimental trials) and not every subject exhibited this gaze pattern (18 subjects looked back to the located object on less than 5 trials, average = 3.2).

### Discussion

The results from the response time analysis replicated the congruence effect [27] with shorter response times for matching than mismatching picture-sentence pairs. Reaction-time analyses also replicated the advantage for ‘above’ compared with ‘below’ trials [43]. Thus, the paradigm can capture established phenomena in spatial language apprehension.

The analysis of the eye movements revealed, at least qualitatively, a pattern in line with the predictions of the AVS account. In fact the first inspection after one inspection to the reference object was directed to the located object. However, this specific re-inspection pattern occurred on only 21.8 percent of the experimental trials, suggesting that such an overt attention shift is not mandatory for understanding and verifying spatial relations. The high accuracy (3.1% incorrect responses) further corroborates this view.

The relatively small number of trials on which participants shifted their eye gaze from the reference object to the located object could result from the task (i.e., verification was speeded and people had little time to execute this gaze shift). In order to examine the role of task constraints and to see whether we can replicate the results from Experiment 1, participants were instructed to respond after the presentation of the sentence in Experiment 2.

## Experiment 2

### Method

#### Participants

Thirty-two further students (average age = 25, range = 20–33) from the University of Bielefeld received five euro each for taking part in this study. All participants had normal or corrected-to-normal vision, were native speakers of German and monolingual before age 6.

Materials, design, and analyses were identical to Experiment 1. The procedure was identical to Experiment 1 but participants were instructed to respond after the presentation of the sentence in Experiment 2.

### Results

Trials with an incorrect response or with a response prior to sentence offset were removed from the analysis (10.3% of the data; 2.9% errors and 7.4% anticipated responses).

#### Response times

The LMER model revealed a significant main effect of sentence value (t = 5.8, SE = 15.7) with faster responses to matching (M = 5426 ms; SD = 573 ms) than mismatching trials (M = 5609 ms; SD = 545 ms). No other effects were significant. As expected, response times were descriptively slower in Experiment 2 than 1 (by 382 ms, i.e., people waited until sentence end before responding).

#### Eye movements

The linear model revealed that the intercept (Estimated coefficient_ss_ = 0.84, t = 2.46, p = <.05; Estimated coefficient_item_ = 0.81, t = 1.99, p < .05) was significant and the positive sign indicated a preference for looking at the located object (see Fig. 4). The analyses on the number of trials showed that, unlike in Experiment 1, participants looked back from the anticipated reference object to the located object on 453 out of 495 correct experimental trials with little variation across participants. Thus, this eye gaze behavior increased from 21 percent in Experiment 1 (when participants performed speeded, on-the-fly responses during sentence comprehension) to 91 percent in Experiment 2 (when responses were given post-sentence).

### Discussion

The analysis revealed faster responses for matching than mismatching trials, replicating the results from Experiment 1. The advantage for ‘above’ trials was not replicated. This effect was originally explained in terms of the strategy participants used to scan a picture [43]. People tend to explore a scene from the top to the bottom, leading to faster verification of ‘above’ than ‘below’ relations. In Experiment 2, participants had more time for the post-sentence verification task than in Experiment 1 (speeded verification). They may have used this extra time to re-explore the scene in a different object order (for example from the bottom to the top), eliminating the response time advantage.

Just as in Experiment 1, the log-gaze probability ratio analyses provided support for the AVS account. The likelihood of inspection to the located object increased after the onset of the spatial preposition and after one inspection to the second-named reference object (see Section B in S1 File). This gaze pattern emerged on 91 percent of 495 correctly answered experimental trials, and thus much more often than in Experiment 1 (21.8 percent of trials with a correct response). The reverse gaze shift thus emerges robustly if comprehenders have sufficient time.

While the reverse shift from the second to the first-named object emerges robustly in Experiment 2 under relaxed time constraints, it is by no means mandatory. Under high time pressure it occurred on only few trials, and error rates did not increase when the gaze shift was infrequent (Experiment 1). We think that participants used the extra time in Experiment 2 to look back at the located object, perhaps to double check the scene before the response. Interestingly, the timing of the gaze shift on a trial did co-vary with that trial’s verification time (an earlier shift back to the located object coincided with a shorter verification time for that trial, see the General Discussion and Fig. D in S1 File).

It has been shown that the task can modulate a perceiver’s gaze pattern [44]. To ascertain the task specificity of the observed gaze pattern (see [45]), we employed a passive-listening task in Experiment 3. Replicating the results from Experiments 1 and / or 2 would suggest that task plays no substantial role. By contrast, clear changes in the gaze pattern for a passive-listening task would corroborate the view that task can modulate the deployment of overt visual attention shifts during spatial language processing (see also [46]).

## Experiment 3

### Method

#### Participants

A further 32 students (average age = 23.8, range = 19–30) from the University of Bielefeld received four euro each for participating in this study. All participants had normal or corrected-to-normal vision and all were native speakers of German and monolingual before age 6.

Materials, design, and analyses were identical to Experiment 1. The procedure was identical to Experiment 1 but no response was required. As a consequence, scene presentation duration was calculated based on the average response time in Experiment 1. We asked participants to look attentively at the scene as they listened to the spoken sentence.

### Results

The analyses on inspection probabilities revealed a significant positive intercept (Estimated coefficient_subj_ = 2.22, t = 3.06, p < .001; Estimated coefficient_item_ = 2.37, t = 3.12, p < .001). As before, people preferred to inspect the located object after one inspection to the reference object and after the onset of the spatial preposition (see Fig. 4). However, this gaze shift occurred only on 138 trials (27.9%), thus mirroring the low frequency of this gaze shift in Experiment 1. Moreover, all participants but one looked back to the located object at least once, but the majority showed this behavior on only one or two experimental trials.

### Discussion

The analyses of inspections replicated participants’ tendency to look back at the located object in line with the AVS-based predictions (see Experiments 1 and 2) but just as in Experiment 1, this behavior was infrequent. The infrequent attentional shifts occurred during speeded, on-the-fly picture-sentence verification (Experiment 1) and in passive listening (Experiment 3) and thus at least this aspect of the eye-movement record appears to generalize across verification and passive listening (see [20]).

In summary all three experiments provide support for the conclusion that an overt visual shift from the reference object (the sausage) to the located object (the box) occurs but is not essential for apprehending the kinds of spatial relations that we examined. The low number of errors in the verification task even when these shifts were infrequent (as was the case in Experiment 1) further corroborates this view. And yet these shifts were present on a small number of trials in all three experiments. This raises the possibility that they may have contributed at least somewhat to the comprehension of the spatial description. If this were the case, then people’s comprehension of the spatial relations should be negatively affected if we prevent these shifts altogether. This was achieved by introducing a gaze-contingent manipulation (e.g., [47], [48]). We removed the (first-mentioned) located object after a participant’s gaze had shifted away from it. The idea behind this manipulation was that people should prefer to look at “something” (i.e., the remaining reference object) rather than at empty space (the location where the located object had been before its removal).

If these shifts back to the located object contribute to the comprehension and verification of the spatial description (as measured by response times and accuracy in the verification task), then we should see a decrease in accuracy and an increase in response times when we discourage participants from executing these shifts by removing the located object. Alternatively we should replicate similar response times and accuracy as in the first two experiments, indicating that these shifts do not play a substantial role for post-sentence verification of spatial relations.

## Experiment 4

### Method

#### Participants

Thirty-two further students (average age = 24, range = 18–32) from the University of Bielefeld received five euro each for taking part in this study. All participants had normal or corrected-to-normal vision, were native speakers of German, and monolingual before age 6.

The 32 critical stimuli were identical to those for Experiment 1. In contrast with Experiments 1–3, all of the 32 critical item sentences matched the picture and the analyses thus do not include the factor congruence. Instead, we included true/false filler trials (N = 32). A further 28 fillers included different types of sentence structures, spatial prepositions, and references to objects that were not depicted.

The task was the same as in Experiment 1. The design was modified from the previous studies by including one additional factor (object removal). Object removal consisted in removing two of the three objects during sentence presentation: in half of the critical trials we removed the reference object and the competitor (the sausage and the walnut in Fig. 1) and in the other half we removed the located object and the competitor (the box and the walnut in Fig. 1). The rule that controls object removal was gaze-contingent (see [47], [48]). The rule triggered only if at least one fixation had been directed to the located object within the critical interval. Otherwise, all the objects remained on the screen. Trials for which the rule did not trigger were eliminated from further analyses. The rule applied in the interval between the onset of the spatial preposition (+200 ms) and the response when a participant’s eyes left the located object. For example, given the sentence ‘The box is above the sausage’, after fixating the box and hearing the beginning of the spatial term, one of two things could happen: (1) either the potential upcoming referent, (e.g., the sausage) and the competitor (e.g., the walnut) disappeared as soon as the eyes moved away from the located object (the box); or (2) the located object and the competitor were removed as soon as the eyes moved away from the located object. The first removal-condition (reference object and competitor) was intended to discourage participants from anticipating the reference object. The second removal-condition (located object and competitor) discouraged participants from re-inspecting the located object after their eye gaze had shifted away from it. The latter eye-gaze removal should thus prevent the overt shift expected based on the AVS model while the former should prevent the anticipatory shifts predicted by the visual-world studies.

#### Procedure

The procedure was identical to Experiment 1 but the trial structure included the object removal factor (see above). After the experiment, participants were asked to identify the research question and the critical manipulation; nobody guessed that the object removal was gaze-contingent or identified the goal of the experiment.

### Analysis

#### Response times

We included only critical trials for which the gaze-contingent removal of the objects had occurred and for which the response was correct. Response time distributions were modeled with Linear Mixed Effects Regression (LMER) including spatial preposition and object removal as independent variables. As before, the predictors were centered. In order to evaluate whether the object-removal manipulation made the task overall more difficult, we compared response times from Experiment 1 and Experiment 4 (correct trials only).

#### Eye movements analysis

Trials on which object removal did not occur (10% of the correct trials) were removed before submitting the data to the model. We also did not include trials for which the object was removed after the response button press (48%). As the number of fixations directed to the competitor object or to the background was very low (< 5%), we took into account only fixations towards the located object and the reference object.

We first verified whether the object removal manipulation was successful. If successful, people should not look back to the location of the located object when that object had been removed, and they should not anticipate the reference object when the reference object had been removed. This should lead to an interaction of object-directed fixations with object removal condition. We analyzed object fixations with a generalized mixed-effect model for data with a binomial distribution [33]. We included spatial preposition and object removal as fixed factors, and subject and item as random-effect factors (see Table A in S1 File).

#### Performance (error distribution analysis)

In order to evaluate whether the looks back to the located object contribute towards the comprehension and verification of spatial relations, we compared the number of errors between trials in which participants did not look back at the located object, and trials in which the removal did not occur (and for which people were able to inspect the located object). This provided insight into the overall effect of object removal on performance. Furthermore, on trials where the removal did occur, we compared the error number for the condition in which the *located* object was removed with the condition in which the *reference* object was removed. This permitted us to compare performance when participants did not overtly shift back to the located object relative to when they did not anticipate the reference object. As above, we used a generalized mixed-effect model for data with a binomial distribution.

### Results

Trials with an incorrect response (4.25% of the data) were removed from the analysis.

#### Response times

The LMER model revealed a significant effect of spatial preposition (t = -3.05, SE = 52.2; M*above* = 4462 ms; M*below* = 4606 ms) but not of object removal (located object and competitor vs. reference object and competitor, n.s.). Statistical comparison with Experiment 1 revealed a significant difference (t(62) = 4.45, p < .0001) with faster reaction times for Experiment 4 (M = 4462 ms) than for Experiment 1 (M = 5013 ms).

#### Eye movements

The generalized mixed-effect model showed a significant effect of object removal (t = 13.6, SE = 0.16). Participants mostly fixated whichever object remained on the screen (the located object when the reference object and the competitor had been removed; the reference object when the located object and the competitor had been removed).

#### Performance (error distribution)

There was no reliable difference in the number of errors between trials for which the removal occurred (1.6% errors) and trials for which the removal did not occur (2.2%, t = 0.02, SE = 0.48). The GL model on trials where the removal occurred revealed no significant difference in performance for either the spatial preposition (t = -0.69, SE = 0.43) or the object removal factor (t = -0.65, SE = 0.41).

### Discussion

In the eye-gaze analysis, for almost half of the trials (48%) the gaze-contingent change did not occur, meaning that people did not anticipate the reference object following the spatial term. For our question, the trials of interest are those for which the gaze-contingent object removal *did* take place. For those trials, a main effect of object removal resulted from a higher number of trials on which participants preferred to look at whichever object remained on-screen. Overall, people shifted back to the located object on 34 trials only (1.2%). The object removal thus succeeded in preventing people from shifting back to the located object, a behavior affecting neither response times nor accuracy.

If removing the located object (and thus preventing its re-inspection) had affected the verification performance negatively, then accuracy should have been reduced in this condition relative to the condition in which the reference object had been removed. However, accuracy and response times were similar between these two object-removal conditions, corroborating that the re-inspection of the located object is not critical for verification performance. Thus in its overt visual form (of an eye movement), the attentive mechanism described in the AVS model [24] does not contribute substantially to verifying and understanding spatial relations as measured by post-sentence response time and verification errors.

## General Discussion

In four experiments, we monitored people’s eye movements while they listened to a spatial description and inspected corresponding clipart pictures. Across the four experiments we varied the task (Experiment 1: speeded verification; Experiment 2: post-sentence verification; Experiment 3: passive listening) and within-experiment we varied object presence (Experiment 4). These changes together with novel, gaze-contingent analyses of participants’ eye-movement behavior permitted us to gain more in-depth insights than previous studies into the role of gaze shifts to objects during spatial language comprehension.

### The AVS Model

After people had heard the spatial preposition and inspected the second-named reference object once, they shifted attention from the reference to the located object—in line with the predictions motivated by the Attention Vector Sum model. However, the frequency of these overt attention shifts was influenced by the task and by object presence. It was infrequent in speeded, on-the-fly verification (Experiment 1), passive listening (Experiment 3), and when the located object was removed (Experiment 4); but it was frequent in post-sentence verification (Experiment 2). Preventing participants from executing these shifts (by removing the located object, Experiment 4) affected neither verification times nor accuracy.

These overt attentional shifts occurred only frequently when people had some “extra-time” and when the task was verification (Experiment 2). If these shifts contributed substantially to verification success then accuracy in the verification task should have been higher when these shifts were frequent. However, this was not the case, and error rates were descriptively similar across the three verification experiments (Exp 1 = 3.1%, Exp 2 = 2.9%, Exp 4 = 4.25%) while the frequency of the AVS-motivated shifts varied among these studies. It is possible that performance was at ceiling (mean accuracy was high in all experiments), and that such shifts would contribute more towards performance in difficult tasks (e.g., when sentences are difficult or the visual context is more complex than in our experiments). But at least for the contexts in our studies, the overt shift from the reference to the located object was not mandatory, and its absence impaired neither the time nor the accuracy of verifying a spatial description against an object depiction.

The comparison between Experiments 1 and 3 revealed that the type of task (verification vs. passive listening) did not modulate the frequency of eye movement shifts from the reference object to the located object. Indeed, these shifts were infrequent in both of these studies (< 30% of the trials). The lack of a clear task effect on eye-movement pattern (Experiments 1: speeded verification vs. Experiment 3: passive listening) is consistent with previous findings (e.g., [20]). It is also in line with the claim that verification is part and parcel of language comprehension (i.e., the assumption is that people perform verification to some extent even when the task does not explicitly require them to do so, see [49], [50], [51]).

At the same time, the frequency of trials on which the AVS-motivated shift occurred, varied substantially between Experiments 1, 3, and 4 (speeded, on-the-fly verification and passive listening tasks) compared with Experiment 2 (delayed, post-sentence verification). Thus, the time allocated for verification does modulate the occurrence of eye gaze shifts from the reference to the located object. Interestingly, the timing of this shift on a given trial correlated with the verification response time for that trial in Experiment 2 (t(274) = 6.5, p < .001, r = 0.367) as well as in Experiment 1 (t(106) = 7.49, p < .0001, r = 0.59) (see Fig. D in S1 File). Thus, more time to verify the spatial relation increased the frequency of these gaze shifts and the time of the gaze shift co-varied with the response time. Future research is necessary to ascertain how precisely the timing of these shifts impacts verification time. To the extent that they contribute robustly to the timing of verification and comprehension processes, models of real-time visually situated language comprehension will want to accommodate them. Likewise, for the Attention Vector Sum model [24] to make clearer predictions for spoken language processing, it would have to be extended by specifying when in time during a sentence the shift occurs, under which conditions it is overt (vs. covert), and whether it could also operate on the mental representations of objects that are no longer present.

### Caveats

One concern is that the attentional shift implicated in apprehending the spatial relation could be performed on the spatial representation built before sentence comprehension (our studies included a preview time). Note however, that our experiments were balanced such that predicting which objects the sentence would be about or predicting specific spatial relations (above versus below) was not possible prior to the sentence. Filler trials included negated sentences (e.g., ‘The sausage is not above the box’), descriptions of objects that were not in the scene and different spatial prepositions (e.g., “zwischen”, ‘between’; “nahe bei”, ‘near’, and “um … herum”, ‘around’). Half of our experimental trials described the middle object out of three objects as located above another object, and the other half described the middle object as located below another object. As a result it was not clear whether the box and the sausage were best encoded as box-above-sausage or box-below-walnut. Arguably then, the apprehension of the described spatial relations and the expected shift could only occur during or after sentence comprehension. In addition, if participants were able to gather all the information necessary to accomplish the task during the preview, eye movements during comprehension would be unnecessary. But it has been suggested that people use eye movements to reduce memory load, and inspect objects “just in time” [52], [53]. This claim fits well with the fixation distribution in our experiments (see Figs. A, B and C and Tables B, C and D in S1 File) for which people’s eye movements were tightly linked to the linguistic input. This suggests that the spatial relation of a sentence was processed once it was mediated by the spatial preposition, but not before.

A further concern is that the critical attentional shift might be performed covertly rather than overtly, which eye movements would not reveal. Objects in our studies were located closely together such that covert attention shifts could, in principle, occur even when no overt shift was measured. People are able to covertly shift their attention away from the target of a saccade in the pause between one saccade and the next [58], [59], [60]. However, the studies that report a spatial dissociation of covert and overt attention differ substantially from our experiments. In the former, participants were instructed to either visually track a flashing dot or to count dots [58], [59], [60]. During this visual task, they were instructed to covertly detect / discriminate a target in a different location. By contrast, in the present studies, participants were not required to discriminate a target at a location away from the gaze focus. It is unclear whether—without this explicit instruction and in a task that foregrounds language processing—people would be likely to split their covert and overt attention.

Moreover, it is largely accepted that covert attention and overt attention are tightly connected as covert shifts always precede overt attentional shifts [54], [55], [56]). It has been claimed that “it is not possible to decide to look at one target while simultaneously and successfully shifting complete perceptual attention to another” ([57], p. 1470). Thus, if people execute a saccade towards the reference object, they cannot—at the same time—direct a covert shift towards the located object. Covert shifts away from the gaze focus are all the more unlikely since participants’ attention was guided by a single task, viz. sentence verification. Indeed, it is possible that the close temporal coupling of covert followed by overt gaze shifts yielded the reliable correlations with verification times that we observed in Experiments 1 and 2. Overall, it is somewhat unlikely that covert shifts were uncoupled from overt gaze shifts, and that such uncoupled covert shifts are responsible for the successful verification of spatial relations in our task.

### Incremental Interpretation (visual-world studies)

Results from the processing of spatial prepositions in visual-world studies (e.g., [11]) do not predict the observed re-inspection of the located object (Experiment 1, 2 and 3). And indeed, incremental interpretation processes (of ‘The box is above…’) best accommodate participants’ eye-movement averages (anticipating the sausage before its mention—see Tables B, C and D in S1 File for details). The gaze averages can be accommodated by existing accounts of situated language processing (e.g., [61]) although these accounts would need to be extended with a more detailed attentional mechanism to capture the (infrequent) looks back to the located object. Attention models by Itti and Koch [62] or the theory of visual attention [63] would need to be extended with a language-processing component to accommodate our findings and with a more specified attentional response to spatial language. Others (e.g., [64]) have presented a framework for language-vision interaction but not a processing account of situated language (i.e., they would not predict the shift back to the located object either and are procedurally underspecified).

An interesting question is whether the eye movement pattern observed in our study is specific to spatial language comprehension or not. We currently examine whether these results generalize to sentences about non-spatial relations between two objects (e.g., “Die Kiste ist größer als die Wurst”, ‘The box is bigger than the sausage’). Failure to replicate would indicate that these shifts are specific to spatial language processing. By contrast, replicating the (albeit infrequent) re-inspection of the located object (the box) following ‘bigger’ would suggest that such (rare) re-inspections are not only implicated in processing spatial language, but in establishing all sorts of relations between objects. Finally, another possibility is that these shifts against the grain of the utterance occur even for non-relational utterances (e.g., when the objects are enumerated).

In conclusion, the present study uncovered for the first time the overt attentional shifts during spatial language comprehension. Our findings are important for spatial language understanding and highlight the need for a more explicit account of the visual attentional behavior during, and its functional contribution to, language comprehension. The results also highlight the need for a better specification of the attentional mechanism described in the AVS model, given that the current version is not able to accommodate the outcomes from our experiments.

## Supporting Information

S1 File **Section A,** Summary of the mixed models (LMER) and linear models (lm) used throughout the paper. **Section B,** Integrative analysis of eye movements based on the percentage of fixations and log-gaze probability. **Section C,** Time-course of spatial description understanding based on the log-gaze probability of fixations. **Section D,** Correlation between gaze shift time and response latencies for Experiment 1 and 2. **Section E,** List of sentences used in the experiments.
(DOCX)
