# Supplementary material for: Visual Attention during Spatial Language Comprehension
Source: PLoS One. 2015 Jan 21;10(1):e0115758. doi: 10.1371/journal.pone.0115758 (PMC4301815; doi:10.1371/journal.pone.0115758)
Supplement: S1 File — Section B, Integrative analysis of eye movements based on the percentage of fixations and log-gaze probability. Section C, Time-course of spatial description understanding based on the log-gaze probability of fixations. Section D, Correlation between gaze shift time and response latencies for Experiment 1 and 2. Section E, List of sentences used in the experiments. (DOCX) [file pone.0115758.s001.docx]

**Section A**

Table A: Summary of the mixed models (LMER) and linear models (lm) used.

| **Analysis** | **Model** |
| --- | --- |
| *Experiment 1*  - Response time  - Eye movements | lmer (RT ~ SP*SV + (1+ SP+SV\|ss) + (1+ SV\|item))  lm (LogP ~ Fix) |
| *Experiment 2*  - Response time  - Eye movements | lmer (RT ~ SP*SV + (1+SV\|ss) + (1\|item))  lm (LogP ~ Fix) |
| *Experiment 3*  - Eye movements | lm (LogP ~ Fix) |
| *Experiment 4*  - Response time  - Eye movements  - Accuracy | lmer (RT ~ SP*OR + (1\|ss) + (1\|item)  lmer (NrFix ~ OR + (1\|ss) + (1\|item), family = “binomial”)  lmer (acc ~SP+OR + (1\|ss) + (1\|item), family = “binomial”) |

Note: For the mixed models we report the simplified converging model obtained by removing variables that did not contribute significantly towards explaining the variance in the model (model comparison was done using log-likelihood ratio).

Legend: SP = spatial prepositions (“über” vs. “unter”, ‘above’ vs. ‘below’); SV = sentence values (‘true’ vs. ‘false’); LogP = log-gaze probability; Fix = fixations; nrFix = number of fixations; RT = reaction times; acc = accuracy; OR = object removal.

**Section B**

For all the analyses on the eye movement pattern we coded four word regions for each sentence: *NP1* (M_duration_ = 1078), *Verb* (M_duration_ = 378), *Spatial Preposition* (M_duration_ = 606), *NP2* (M_duration_ = 1039). We calculated these regions on a trial-by-trial basis. A first analysis examined the percentage of fixations to the objects for each participant averaged within 100 ms time bins. This reflects the time course of visual attention across the sentence. A second analysis looked at log-gaze probability. We computed mean log gaze probability ratios for the located object (LO) against the gaze probability to look at the reference object (RO) using the following formula: *ln*(P(LO)/P(RO)). Preliminary analyses showed that neither looks to the competitor nor the background affected the log gaze probability pattern; for this reason these regions were not included in the calculation. A zero value indicates that the located object and the reference object received the same amount of attention; a positive score indicates that the located object is inspected more and a negative value indicates that the reference object receives more attention. The sign of the value expresses which object people are more likely to inspect, and the absolute value of the log-ratio indicates the magnitude of the effect. A significant intercept in the linear model indicates that the log-ratio is different from zero, and its valence reveals a preference for looking more towards one of the two objects. When the nominator in the log-ratio formula was 0 (meaning there was no fixation to the reference object), we added a constant to both the nominator and denominator to enable division.

**Experiment 1**

Table B presents an overview of the fixation percentages on the 4 AoIs (Located object, Reference object, Competitor and Background) within the four critical time intervals; *NP1, Verb, SP, NP2*. In the NP1 time window, fixations were mostly directed towards the located object (the box in the sentence ‘The box is above the sausage’). As participants heard the preposition (SP), they began to shift their attention to the reference object and continued to do so throughout the second noun phrase (NP2). However, fixation proportions to the located object stayed high (relative to the competitor) until the time of the response.

Table B: Percentage of fixations towards the Areas of Interest for each critical time region in Experiment 1. Each row sums to 100 percent.

|  | Located Object (NP1) | Reference Object (NP2) | Competitor | Background |
| --- | --- | --- | --- | --- |
| NP1 | 53 | 20 | 23 | 4 |
| Verb | 49 | 20 | 24 | 7 |
| SP | 34 | 44 | 15 | 7 |
| NP2 | 32 | 54 | 8 | 6 |

Figure A (Section C) illustrates the log-gaze probability calculated for slices of 100 ms from scene onset to the response. It corroborates the results from the fixation percentages but also adds information. As the first object (the box) was named (NP1) participants were more likely to inspect it than the sausage. During the preposition, they were more likely to shift their attention to the sausage. After NP2 offset there was a rise in gaze probability for the located object (the box) followed by a rise in gaze probability for the reference object (the sausage). For the NP1 region, the intercept was significant, revealing more looks towards the located than the reference object (Estimated coefficient_ss_ = 0.697, t = 3.52, p < .001; Estimated coefficient_item_ = 0.536, t = 3.21, p < .01). For the Verb interval, the intercept was not significant (Estimated coefficient_ss_ = 0.173, t = 0.426, p = n.s., Estimated coefficient_item_ = 0.352, t = 0.852, p = n.s), indicating that the two target objects were fixated equally often. For the SP interval, the intercept coefficient for the log gaze probability was significant (Estimated coefficient_ss_ = -2.06, t = -8.98, p < .0001, Estimated coefficient_item_ = -2.02, t = -8.75, p < .0001) and the negative value indicates a preference to look at the reference object. Finally, the intercept for NP2 was also significant (Estimated coefficient_ss_ = -2.06, t = -8.56, p < .0001, Estimated coefficient_item_ = -1.74, t = -6.62, p < .0001) and the negative sign indicates again a higher probability to fixate the reference object.

**Experiment 2**

As for Experiment 1, we provide an overview of the gaze pattern (see the fixation percentages in Table C). During NP1 most fixations were directed towards the located object. As soon as participants heard the preposition (SP), they began to shift their attention to the reference object and continued to do so until the second noun phrase (NP2). Similarly to what we observed in Experiment 1, fixation proportions to the located object stayed high (relative to the competitor) until the time of the response.

Table C: Percentage of fixations towards the four Areas of Interest for each critical time region in Experiment 2. Each row sums to 100 percent.

|  | Located Object (NP1) | Reference Object (NP2) | Competitor | Background |
| --- | --- | --- | --- | --- |
| NP1 | 51 | 22 | 22 | 5 |
| Verb | 50 | 21 | 23 | 6 |
| SP | 33 | 45 | 15 | 7 |
| NP2 | 27 | 58 | 9 | 6 |

The analysis of log-gaze probabilities in the NP1 region revealed a significant intercept and thus main effect of object (Estimated coefficient_ss_ = 0.813, t = 3.576, p < .001; Estimated coefficient_item_ = 0.62, t = 3.87, p < .0001). This was also true for the SP (Estimated coefficient_ss_ = -1.995, t = -9.08, p < .001, Estimated coefficient_item_ = -1.79, t = -8.55, p < .0001) and the NP2 interval (Estimated coefficient_ss_ = -1.5, t = -7.635, p < .0001; Estimated coefficient_item_ = -1.23, t = -5.26, p < .0001) but not for the verb region (Estimated coefficient_ss_ = -0.225, t = 0.593, p = n.s., Estimated coefficient_item_ = 0.245 t = 0.716, p = n.s.). The intercept sign indicated a prevalence of looks towards the located object (NP1) followed by a prevalence of looks to the reference object (SP and NP2) (see Figure B).

**Experiment 3**

As the located object was mentioned, participants’ fixations were mostly directed towards it, and the remaining looks were divided between the reference object and the competitor (Table D). This pattern continued during the verb and spatial preposition. Unlike in the previous two experiments, the shift of visual attention to the reference object emerged later, during the second noun phrase (NP2).

Table D: Percentage of fixations towards the designed Areas of Interest for each critical time region in Experiment 3. Each row sums to 100 percent.

|  | Located Object (NP1) | Reference Object (NP2) | Competitor | Background |
| --- | --- | --- | --- | --- |
| NP1 | 48 | 22 | 20 | 10 |
| Verb | 56 | 17 | 19 | 8 |
| SP | 38 | 23 | 28 | 11 |
| NP2 | 25 | 54 | 13 | 8 |

The log-gaze probability illustrated in Figure C shows that as the first object (the box) was mentioned, participants were more likely to inspect it than the other object (the sausage). After hearing the spatial preposition, there was a subtle trend towards anticipating the second-mentioned reference object. At NP2 offset a rise in the probability to inspect the located object emerged. The intercept of the log gaze probability of fixations during NP1 revealed a prevalence of looks towards the located object (Estimated coefficient_ss_ = 3.93, t = 10.1, p < .001; Estimated coefficient_item_ = 4.93, t = 13.4, p < .001). The intercepts for the Verb interval were also significant (Estimated coefficient_ss_ = 3.88, t = 4.73, p < .001, Estimated coefficient_item_ = 4.84, t = 5.93, p < .001), with the positive value indicating again a preference to fixate the located object. The intercept coefficient for the log gaze probability in the SP interval was not significant (Estimated coefficient_ss_ = 0.23, t = 0.49, p = n.s., Estimated coefficient_item_ = -0.07, t = -0.15, p = n.s.) suggesting that after hearing the spatial preposition no immediate preference for one of the two target objects emerged. Finally, the NP2 intercept was significant (Estimated coefficient_ss_ = -2.44, t = -6.73, p < .0001, Estimated coefficient_item_ = -2.3, t = -6.75, p < .0001) and the negative sign indicated a prevalence of looks towards the reference object.

**Section C**


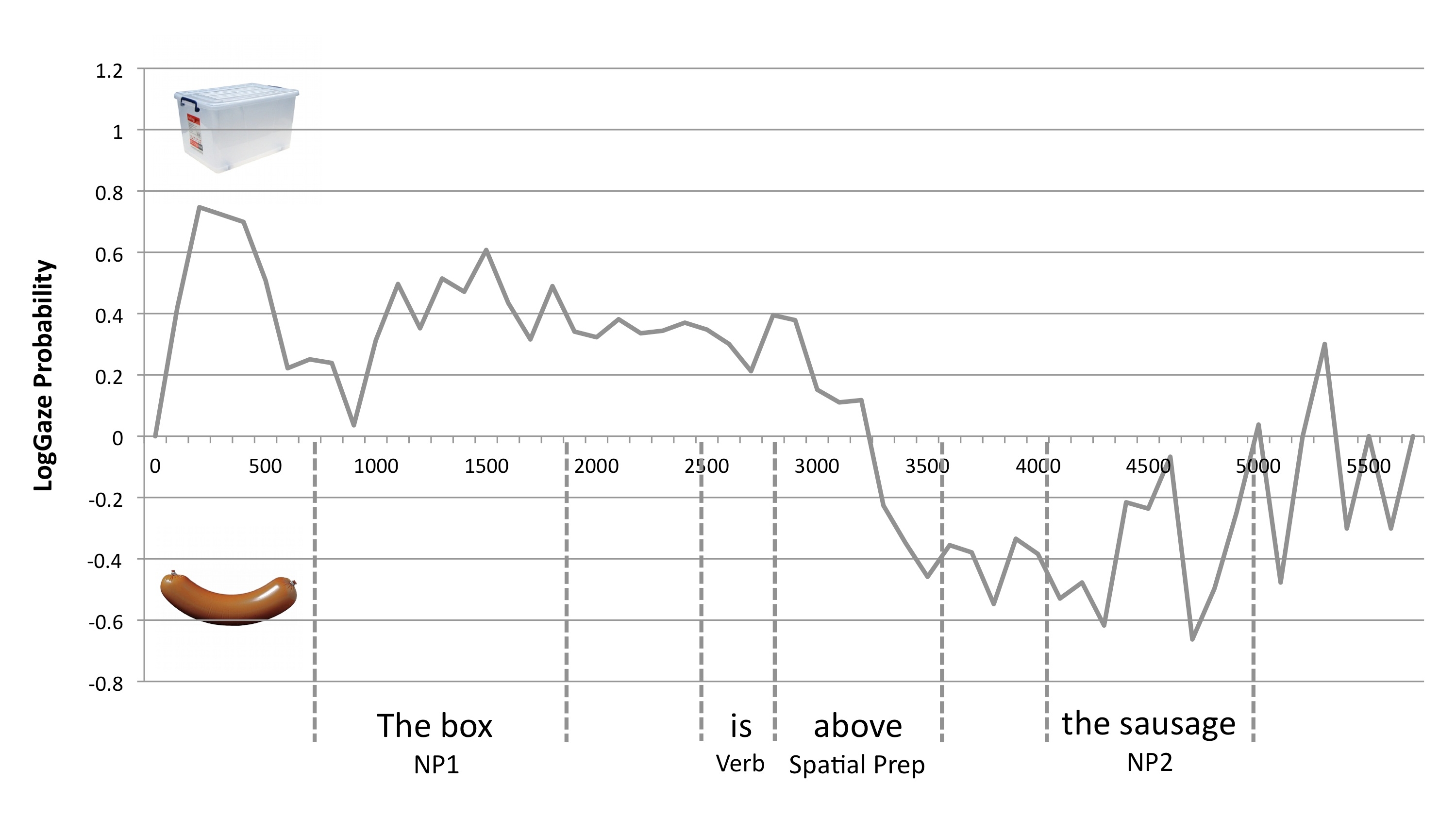


**Figure A: Log-gaze probability of fixation**

Figure A plots the log of the probability of fixating the located object (the box) relative to the reference object (the sausage) in Experiment 1. Positive values indicate a higher probability of looks towards the located object while negative values indicate a higher probability of looks towards the reference object. The time course of the unfolding sentence is plotted on the x-axis. The dotted lines indicate the average word onsets and offsets. The first 750 ms were the preview time.

**
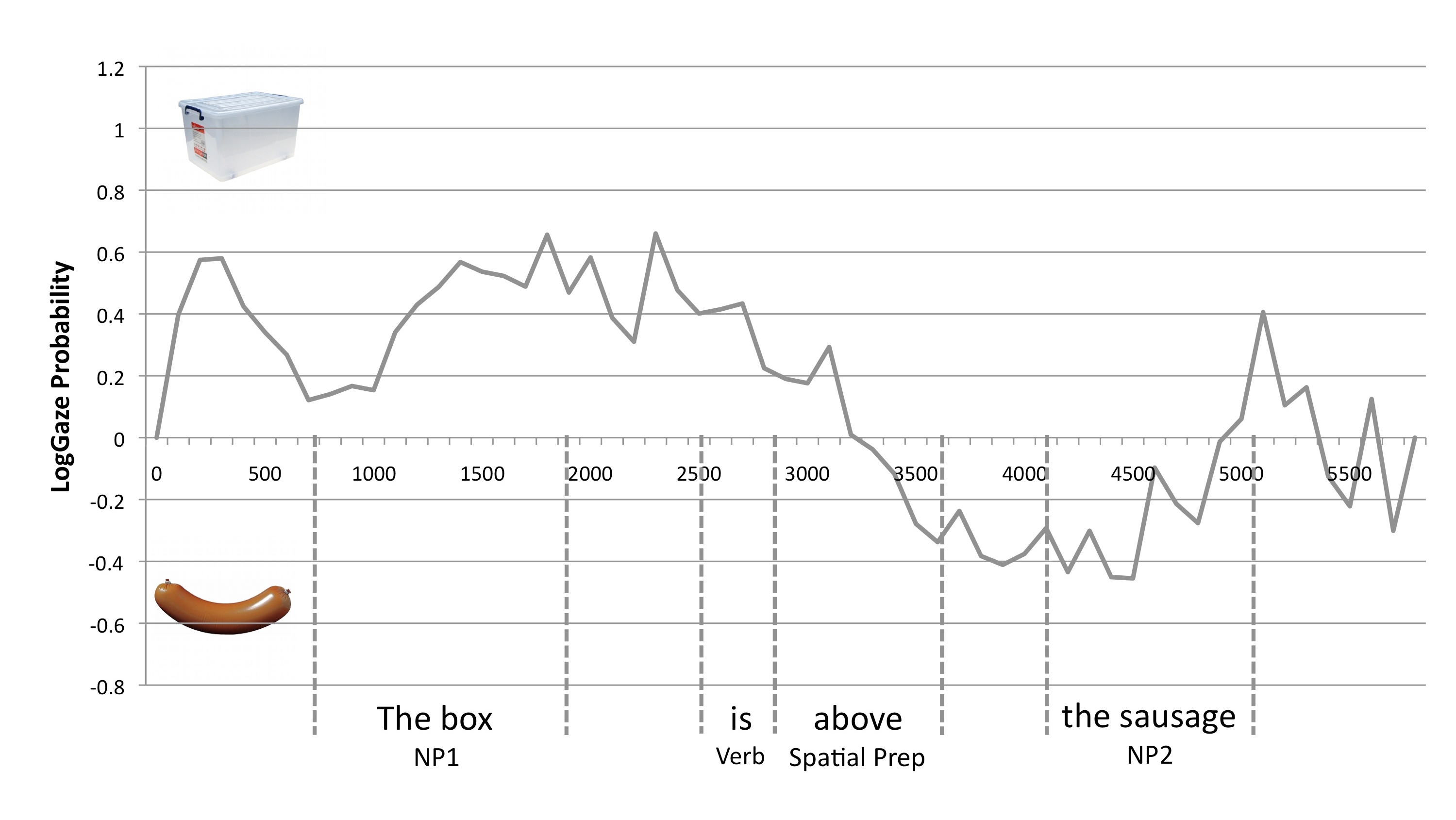
**

**Figure B: Log-gaze probability of fixations**

Figure B plots the log of the probability of fixating the located object (the box) relative to the reference object (the sausage) in Experiment 2. Positive values indicate a higher probability of gazes towards the located object (the box) while negative values indicate a higher probability of looks to the reference object (the sausage). The time course of the unfolding sentence is plotted on the x-axis. The dotted lines indicate the average word onsets and offsets. The first 750 ms were the preview time.


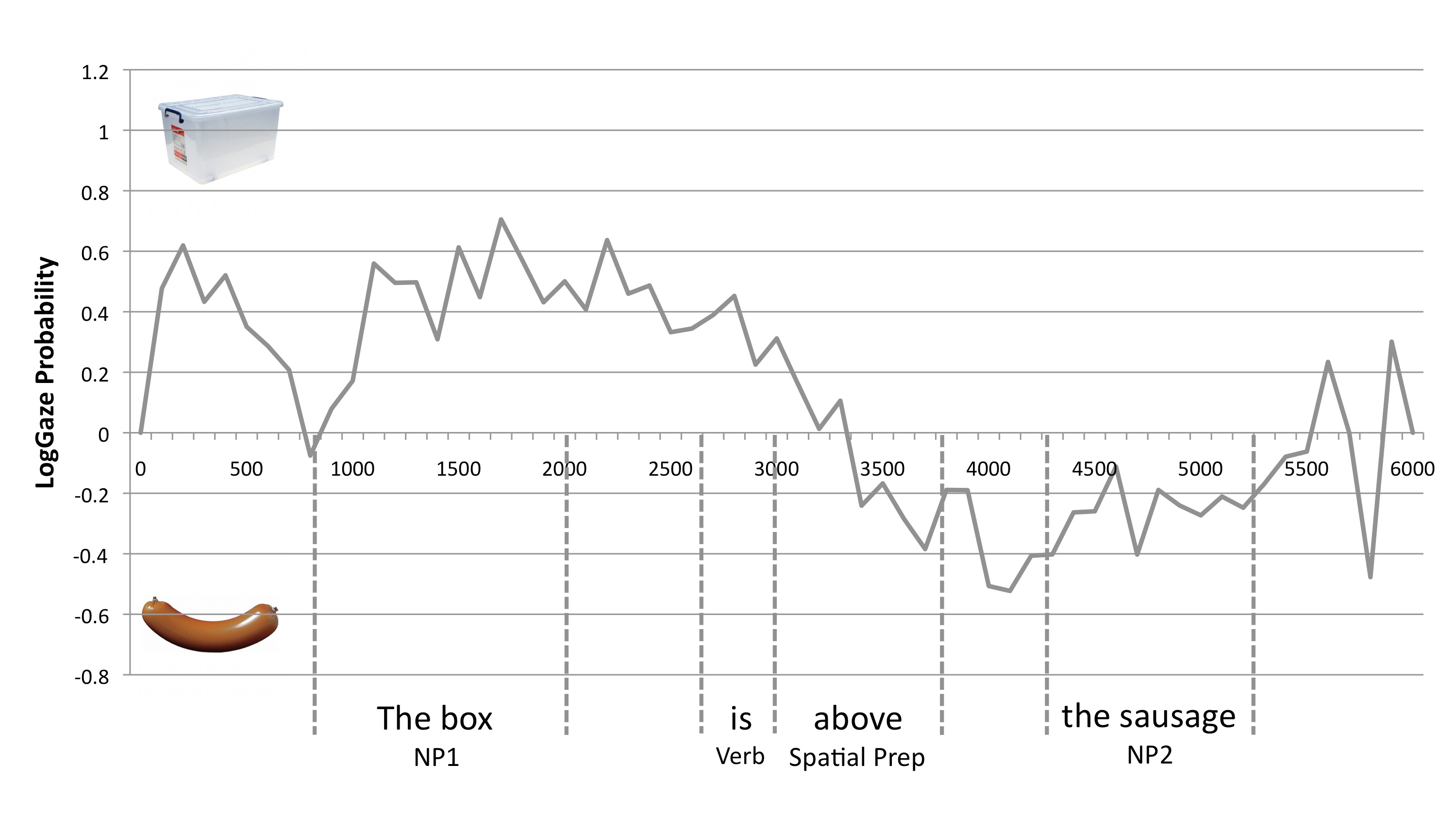


**Figure C: Log-gaze probability of fixations**

Figure C plots the log of the probability of fixating the located object (the box) relative to the reference object (the sausage) in Experiment 3. Positive values indicate a higher probability of gazes towards the located object (the box) while negative values indicate a higher probability to look at the reference object (the sausage). The time course of the unfolding sentence is plotted on the x-axis. The dotted lines indicate the average word onsets and offsets. The first 750 ms were the preview time.

**Section D**

**
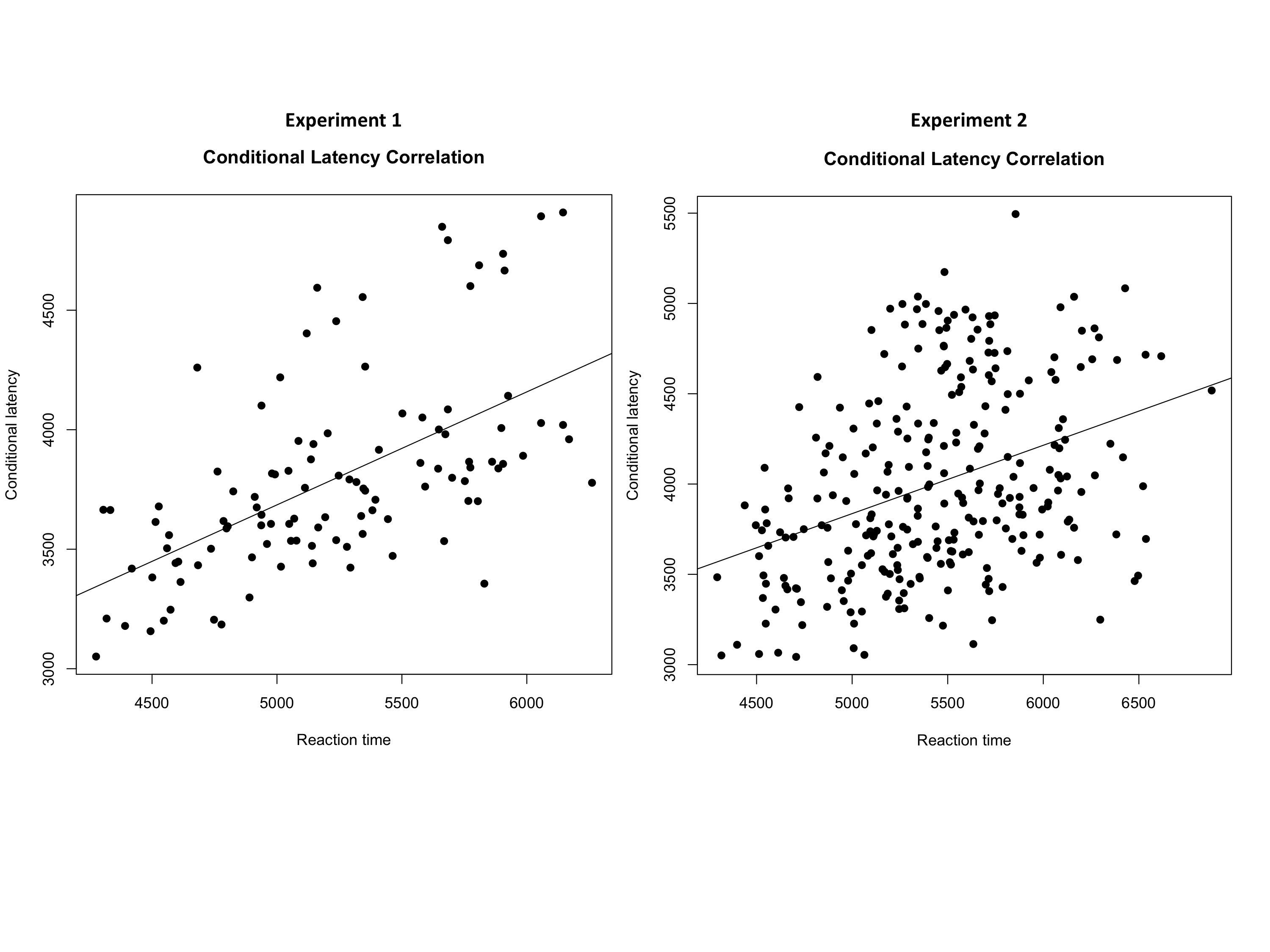
**

**Figure D: Correlations between the time of the AVS-based gaze shifts and the reaction times in Experiment 1 (left) and Experiment 2 (right).**

**Section E**

List of the 32 critical sentences (Experiments 1 to 4).

1) Die Zwiebel ist über / unter der Muschel.

2) Der Würfel ist über / unter dem Kaktus.

3) Der Leuchtturm ist über / unter dem Spiegel.

4) Der Diamant ist über / unter dem Kopfhörer.

5) Das Geodreieck ist über / unter dem Butterbrot.

6) Die Schreibmaschine ist über / unter der Badewanne.

7) Der Kleiderbügel ist über / unter dem Schraubenzieher.

8) Das Paket ist über / unter dem Sofa.

9) Das Fass ist über / unter dem Sieb.

10) Das Lineal ist über / unter dem Funkgerät.

11) Die Zigarre ist über / unter der Kartoffel.

12) Die Pyramide ist über / unter der Büroklammer.

13) Der Fön ist über / unter dem Rock.

14) Der Kamin ist über / unter dem Teller.

15) Der Staubsauger ist über / unter dem Regenschirm.

16) Der Ball ist über / unter dem Hut.

17) Die Mistgabel ist über / unter der Erdbeere.

18) Der Eimer ist über / unter dem Bleistift.

19) Die Box ist über / unter der Wurst.

20) Der Teppich ist über / unter dem Kuchen.

21) Das Gewicht ist über / unter dem Radio.

22) Der Ziegelstein ist über / unter dem Rollator.

23) Der Tennisschläger ist über / unter dem Schraubenschlüssel.

24) Der Koffer ist über / unter dem Hammer.

25) Die Kirsche ist über / unter der Geige.

26) Das Klavier ist über / unter dem Denkmal.

27) Das Karussell ist über / unter dem Taschentuch.

28) Das Blatt ist über / unter dem Bett.

29) Die Banane ist über / unter der Schublade.

30) Der Löffel ist über / unter dem Anker.

31) Die Schaufel ist über / unter der Vase.

32) Die Seife ist über / unter der Tafel
